# Polystyrene Microplastics and Lead Co-Exposure Disturbed Hepatic Lipid Metabolism in C57BL/6 Mice

**DOI:** 10.3390/metabo16050325

**Published:** 2026-05-13

**Authors:** Bei Gao, Mengru Wei, Meng Zhao, Weichen Xu, Guangyuan Liu, Weishou Shen, Pengcheng Tu, Jinjun Shan

**Affiliations:** 1 Jiangsu Key Laboratory of Atmospheric Environment Monitoring and Pollution Control, Collaborative Innovation Center of Atmospheric Environment and Equipment Technology, School of Environmental Science and Engineering, Nanjing University of Information Science and Technology, Nanjing 210044, China; bgao@nuist.edu.cn (B.G.); 20211248134@nuist.edu.cn (M.Z.); wsshen@nuist.edu.cn (W.S.); 2 School of Marine Sciences, Nanjing University of Information Science and Technology, Nanjing 210044, China; 202412371979@nuist.edu.cn; 3 Jiangsu Key Laboratory of Pediatric Respiratory Disease, Medical Metabolomics Center, Institute of Pediatrics, Nanjing University of Chinese Medicine, Nanjing 210023, China; xuweichen@njucm.edu.cn; 4 Department of Radiology, Dalian Public Health Clinical Center, Dalian 116033, China; 18004266581@189.cn; 5 Department of Environmental Health, Zhejiang Provincial Center for Disease Control and Prevention, Hangzhou 310051, China

**Keywords:** microplastics, heavy metal, combined toxicity, lipidome, PS

## Abstract

**Background:** Microplastics and lead are ubiquitous pollutants in the environment, frequently found in soil, water and food. Although the toxicity of individual exposure to microplastics and lead has been well studied, research on their co-exposure effects is still emerging. **Methods:** In this study, we investigated the impacts of polystyrene microplastics and lead co-exposure on hepatic lipid metabolism through transcriptomics and untargeted lipidomics profiling in C57BL/6 mice. **Results:** Gene set enrichment analysis of the transcriptomics data revealed the fatty acid metabolism was significantly disrupted by the co-exposure in male mice. Consistently, pathway analysis of lipidomics data showed that several fatty acid pathways were activated by the co-exposure in male mice, including fatty acid (20:1) → fatty acid (22:1) → fatty acid (24:1), fatty acid (20:0) → fatty acid (22:0) → fatty acid (24:0), fatty acid (18:1) → fatty acid (18:2) → fatty acid (20:2), fatty acid (18:1) → fatty acid (18:2) → fatty acid (18:3) → fatty acid (18:4); meanwhile, fatty acid (18:4) → fatty acid (20:4) → fatty acid (22:4), fatty acid (18:4) → fatty acid (20:4) → fatty acid (20:5) were suppressed. Transition from diacylglycerol to phosphatidylethanolamine and from phosphatidylserine to phosphatidylethanolamine were activated by the co-exposure in male mice; meanwhile transition from phosphatidylserine to phosphatidylethanolamine was activated in female mice. **Conclusions**: Our findings suggested that hepatic lipid metabolism was disturbed by the co-exposure of polystyrene microplastics and lead, which provide insights into the combined exposure risks of microplastics and heavy metals.

## 1. Introduction

Microplastics (MPs), defined as plastic particles with a diameter smaller than 5 mm, are global environmental pollutants. These tiny plastic fragments originate from various sources, such as the breakdown of large plastics, synthetic textile fibers, and microbeads used in personal care products. Due to their small size and environmental persistence, MPs are pervasive in the ecosystem. They have infiltrated air, water, and food chains, making human exposure inevitable [1]. Transfer of MPs from intravenous fluid delivery systems into circulatory systems also contributes to human MP exposure [2]. The emergence of MPs in blood, liver and various human tissues has been reported, raising serious concerns about their health risks [3,4]. The mean value of total MPs detected in decedent human livers reached 433 µg/g [5]. MPs have been considered as a global threat to life [6].

MPs could act as carriers for other toxic pollutants, including heavy metals [7,8]. MPs and heavy metals interact with each other in sediments [9] and aquatic environments [10]. The adsorption of heavy metal lead (Pb) onto polystyrene (PS) MPs is a physical adsorption process and is spontaneous [11]. Pb is a naturally occurring heavy metal in the earth’s crust, as well as a ubiquitous persistent environmental toxicant. Pb can enter the human body through ingestion of contaminated food and water, inhalation of air contaminated with Pb dust, and direct skin contact, which adversely affects multiple organs and causes various diseases [12,13]. Liver serves as the primary target organ where substantial Pb accumulation occurs [14]. Recently, Pb poisoning in preschool children in Gansu Province, China has raised serious concern about the health effects of Pb. The co-existence of Pb and MPs has been detected in aquatic products [15], teabags [16] and even human samples [17]. Their combined toxicological effects have become a public health concern globally.

In our previous study, we showed that Pb and PS MP co-exposure disturbed the gut microbiota and fecal metabolome in C57BL/6 mice [18]. However, the impact of co-exposure on the host hepatic metabolism remains underexplored. Liver damage induced by individual exposure to either MPs or Pb has been documented [19,20], through mechanisms such as the induction of oxidative stress and inflammatory responses [21,22,23]. The liver is rich in endogenous lipids and plays a critical role in lipid metabolism. Disturbance of lipid metabolism by MPs has been previously reported [24,25,26]. However, the effect of MPs and Pb co-exposure on hepatic lipid metabolism remains elusive.

In this study, we investigate the impact of PS MPs and Pb co-exposure on the hepatic lipid using both male and female C57BL/6 mice by integrating transcriptomics and untargeted lipidomic profiling. PS MPs were chosen as they have been reported as one of the most frequently detected MPs in human blood and feces [27]. Our study provides insights into the toxicity assessment of the co-exposure of MPs and heavy metals.

## 2. Materials and Methods

### 2.1. Animals

Specific pathogen free male (*n* = 41) and female (*n* = 42) C57BL/6 mice were used in this study, which were 5-week-old and purchased from Qinglongshan Laboratory (Nanjing, China). Mice were randomly assigned to different cages on a weekly basis for two consecutive weeks. Only mice with the same sex could be co-housed in one cage. When the mice were 7 weeks old, they were randomly divided into four groups: the Ctrl group (*n* = 10–12), PS group (*n* = 10–11), Pb group (*n* = 12), and PbPS group (*n* = 9). Mice were randomly assigned to cages, with 3 to 4 mice per cage. In the PS group, mice were provided with drinking water containing 10 ppm PS. In the Pb group, mice were given water containing 1 ppm PbCl_2_. In the PbPS group, the drinking water was supplemented with both 10 ppm PS and 1 ppm PbCl_2_. The exposure period was 5 weeks. The control group received water without any addition of either PS or PbCl_2_. The mice got access to water and feed *ad libitum*. The size of the PS beads utilized in this experiment was 9–10 µm. The temperature and humidity in the experiment were maintained at 22–25 °C and 40–70%, respectively. The light dark cycle was maintained at 12 h–12 h. The protocol was approved by the Institutional Animal Care and Use Committee (NO. 202139).

### 2.2. Materials and Reagents

Anhydrous PbCl_2_ (99.99%) was obtained from J&K Scientific (Shanghai, China). PS beads (9–10 µm, 2.5% *w*/*v*) were purchased from BaseLine ChromTech Research Center (Tianjin, China). An ALT Activity Assay Kit was obtained from Solarbio Life Sciences (Beijing, China). TRIzol was purchased from ThermoFisher Scientific (Waltham, MA, USA). An NEBNext Ultra RNA library Prep Kit was purchased from New England Biolabs (Ipswich, MA, USA). Methanol (99.9%), ultrapure water, acetonitrile (99.9%) and isopropanol (99.9%) were purchased from ThermoFisher Scientific (Waltham, MA, USA). Toluene (99.9%) and methyl tert-butyl ether (MTBE, 99.8%), were purchased from Sigma-Aldrich (Burlington, MA, USA).

### 2.3. Serum Alanine Aminotransferase (ALT) Measurement

Serum ALT activity, a biomarker of hepatic injury, was measured using an ALT Activity Assay Kit (Solarbio Life Sciences, Beijing, China), following the manufacturer’s protocol. The absorbance was measured at 505 nm using ReadMax 500F microplatereader (Shanghai Kezhe Biochem Technology Co., Ltd., Shanghai, China).

### 2.4. Transcriptomics Sequencing

Total RNA in the liver samples was extracted using TRIzol. The cDNA library was prepared using an NEBNext Ultra RNA library Prep Kit (New England Biolabs, Ipswich, MA, USA) following the manufacture’s instruction. Sequencing was performed by GENE DENOVO Biotech (Guangzhou, China) using Illumina sequencing platform. Trimmomatic-0.39 software was used for quality control with a default setting. Salmon (v.1.8.0) was used for the quantification of transcript expression.

### 2.5. Extraction of Hepatic Lipids

Extraction of hepatic lipids was performed as described in our previous study [28]. Briefly, 10 mg of liver sample from each mouse was extracted with 225 μL of methanol and 750 μL of MTBE. The mixture was subsequently vortexed for 6 min at 4 °C, followed by the addition of 188 μL of ultrapure water. The mixture was then centrifuged at 14,000× *g* for 2 min using Microfuge (Beckman Coulter Life Sciences, Brea, CA, USA). In total, 350 μL of the upper layer was transferred to a 1.5 mL centrifuge tube and evaporated to complete dryness using ZLS-1Vacuum Centrifugal Concentrator (Hexi Instrumentation Equipment Co., Ltd., Changsha, China). A total of 110 μL of methanol/toluene (9:1) solution was added to resuspend the dried sample, which was vortexed for 10 min, followed by 10 min of centrifugation at 18,000 r/min. Finally, 90 μL of the supernatant was collected for further lipidomics profiling.

### 2.6. Untargeted Lipidomics Profiling

Untargeted lipidomics profiling of liver samples was performed as described in our previous study [28]. Briefly, separation of hepatic lipid was performed on Vanquish UHPLC (Thermo Scientific, Waltham, MA, USA) using an Acquity UPLC CSH C18 column (2.1 × 100 mm, 1.7 μm). Mobile phase A consisted of acetonitrile and water (*v*/*v* = 40:60). Mobile phase B contained isopropanol and acetonitrile (*v*/*v* = 90:10). The elution program was as follows: 0–2 min, 15% B; 2–2.5 min, 48% B; 2.5–11 min, 82% B; 11–11.5 min, 99% B; and 11.5–12 min, 15% B. Spectra were collected using Q-Exactive HF (Thermo Scientific, Waltham, MA, USA). Data was collected in both positive and negative electrospray ion source. The mass range *m*/*z* was set from 50 to 1700. Data-dependent mode was used for tandem mass spectrometry (MS/MS) spectra acquisition. Lipidomics raw data files were converted to ABF files by an ABF converter (https://www.reifycs.com/abfconverter/, accessed on 10 May 2024). MS-DIAL was used for data processing and lipid identification as described in our previous study [28].

### 2.7. Statistical Analysis

For RNAseq analysis, DESeq2 was used for the differential gene expression analysis. GSEA was used for the enrichment analysis of gene sets [29]. For lipidomic analysis, Wilcoxon and Kruskal–Wallis tests were used for the comparison between two groups and multiple groups, respectively. FDR was used to adjust multiple comparisons. BioPAN was used for the fatty acid pathway analysis and complex lipid pathway analysis [30]. MetaboAnalyst 6.0 was used for the partial least squares discriminant analysis (PLS-DA) [31]. Significant level was set to *p* < 0.05 unless otherwise specified.

## 3. Results

### 3.1. PS and Pb Co-Exposure Disturbed the Hepatic Transcriptome

**Figure 1:**
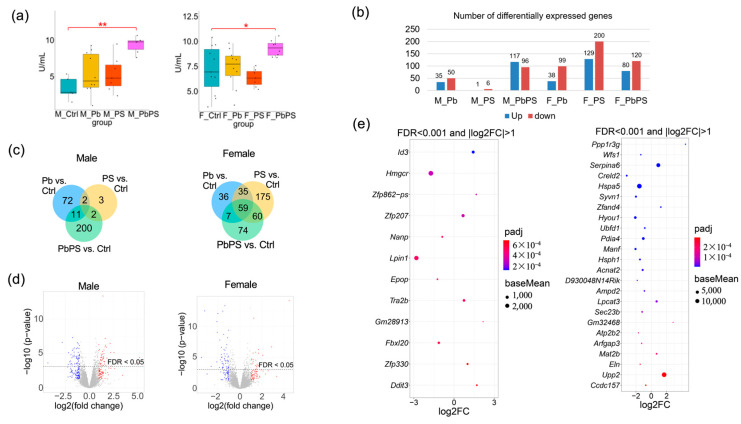
Co-exposure of PS MPs and Pb disturbed the hepatic transcriptome. (**a**) Serum ALT activity (*: *p* < 0.05, **: *p* < 0.01). M: male; F: female. (**b**) Number of differentially expressed genes in exposure groups compared with control group (FDR < 0.05). M: male; F: female. (**c**) Venn diagram of differentially expressed genes in Pb and PS individual exposure groups and PbPS co-exposure group in male ((**left**) panel) and female ((**right**) panel) mice compared with control group (FDR < 0.05). (**d**) Volcano plots of differentially expressed genes in the PbPS co-exposure group compared with controls in male ((**left**) panel) and female mice ((**right**) panel). Red dots: up-regulated genes in co-exposure group with *p*-value < 0.05; blue dots: down-regulated genes in co-exposure group with *p*-value < 0.05; gray dots: not significant. (**e**) Differentially expressed genes in PbPS co-exposure group compared with control group in male ((**left**) panel) and female mice ((**right**) panel) with FDR < 0.001 and |log2FC| > 1. FC refers to fold change.

An increase in serum ALT level was found in PbPS co-exposure group compared with control mice in both males and females, whereas Pb or PS individual exposure did not result in a significant increase in ALT level (Figure 1a). Transcriptomics analysis revealed a number of hepatic genes were differentially expressed (Figure 1b). In male mice, a total of 7, 85 and 213 genes were differentially expressed in the PS and Pb individual exposure groups and the co-exposure group compared with the control group, respectively (FDR < 0.05, Figure 1c, left panel). In female mice, a total of 329, 137 and 200 genes were differentially expressed in the PS and Pb individual exposure groups and the co-exposure group compared with the control group, respectively (FDR < 0.05, Figure 1c, right panel). In the PbPS co-exposure group, 117 up-regulated genes and 96 down-regulated genes were found in male mice; meanwhile 80 up-regulated genes and 120 down-regulated genes were found in female mice compared with the controls (FDR < 0.05, Figure 1d). Differentially expressed genes with FDR < 0.001 and |log2FC| > 1 were shown in the bubble plots (Figure 1e).

### 3.2. Enriched Hepatic Gene Sets in PS and Pb Co-Exposure Group

**Figure 2:**
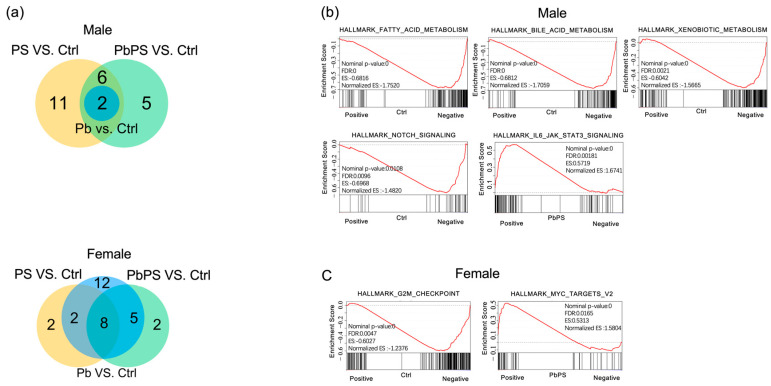
Enriched gene sets by co-exposure of PS MPs and Pb. (**a**) Number of enriched gene sets in exposure groups compared with control group in male ((**top**) panel) and female mice ((**bottom**) panel, FDR < 0.05). (**b**) Significant gene sets altered only by co-exposure instead of individual exposures in male mice (FDR < 0.05). (**c**) Significant gene sets altered only by co-exposure instead of individual exposures in female mice (FDR < 0.05).

GSEA showed that a total of 19, 2, and 13 gene sets were enriched in the PS group, Pb group and the co-exposure group of PS MPs and Pb compared with the control group in male mice, respectively (FDR < 0.05, Figure 2a, top panel). Among 13 gene sets which were significantly altered in the PbPS co-exposure group, five were only enriched by the co-exposure, instead of PS or Pb individual exposure, including the downregulation of fatty acid metabolism, bile acid metabolism, xenobiotic metabolism, NOTCH signaling in the control group, and the upregulation of IL6/JAK/STAT3 signaling in the co-exposure group of PS and Pb (Figure 2b). In female mice, 12, 27, and 15 gene sets were enriched in PS group, Pb group and the co-exposure group of PS and Pb compared with the control group, respectively (FDR < 0.05, Figure 2a, bottom panel). Among 15 gene sets enriched in the PbPS co-exposure group, two were only enriched by the co-exposure, instead of the PS or Pb individual exposure groups, including the downregulation of G2M checkpoint in the control group and the upregulation of MYC targets V2 in the co-exposure group (Figure 2c). These gene sets enriched in males and females were totally different, suggesting the response of liver transcriptome to PbPS was sex-specific.

### 3.3. PS and Pb Co-Exposure Disturbed Hepatic Lipidome

**Figure 3:**
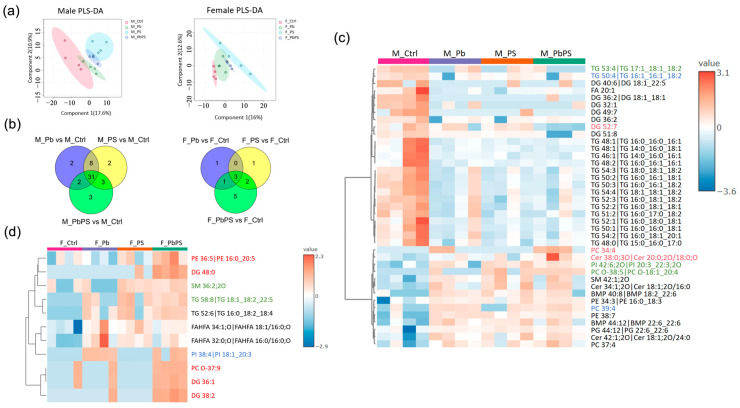
Co-exposure of PS and Pb disturbed the hepatic lipidome. (**a**) PLS-DA plot for male ((**left**) panel) and female mice ((**right**) panel). (**b**) Number of significantly changed lipids in exposure groups compared with control group in male ((**left**) panel) and female mice ((**right**) panel, *p* < 0.05). M: male; F: female. (**c**) Heatmap for metabolites significantly altered in the PbPS co-exposure group compared with the control group in male mice (*p* < 0.05). Black font: metabolites significantly changed in all three exposure groups. Blue font: metabolites significantly changed in both the Pb and PbPS groups. Green font: metabolites significantly changed in both the PS and PbPS groups. Red font: metabolites significantly changed only in the PbPS co-exposure group. (**d**) Heatmap for metabolites significantly altered in the PbPS co-exposure group compared with the control group in female mice (*p* < 0.05). Black font: metabolites significantly changed in all three exposure groups. Blue font: metabolites significantly changed in both the Pb and PbPS groups. Green font: metabolites significantly changed in both the PS and PbPS groups. Red font: metabolites significantly changed only in the PbPS co-exposure group.

Lipidomic profiles of four groups were separated in both male and female mice as shown in the PLSDA plot (Figure 3a). In male mice, there were 43 significantly altered hepatic lipids in the Pb group, 44 in the PS group, and 39 in the PbPS co-exposure group compared with the control group (*p* < 0.05, Figure 3b). These 39 significantly altered hepatic lipids in the PbPS co-exposure group were shown in Figure 3c, among which three lipids were significantly changed only in the PbPS group, instead of the individual exposure groups. In female mice, there were 5 significantly altered hepatic lipids in the Pb group, 6 in the PS group, and 11 in the PbPS co-exposure group compared with the control group (*p* < 0.05, Figure 3b). These 11 significantly altered hepatic lipids in the PbPS group were shown in Figure 3d, among which five lipids were significantly altered only in the PbPS co-exposure group, instead of the individual exposure groups. Stronger responses were observed in male mice than females.

### 3.4. Fatty Acid Pathways Activated by PbPS Co-Exposure

**Figure 4:**
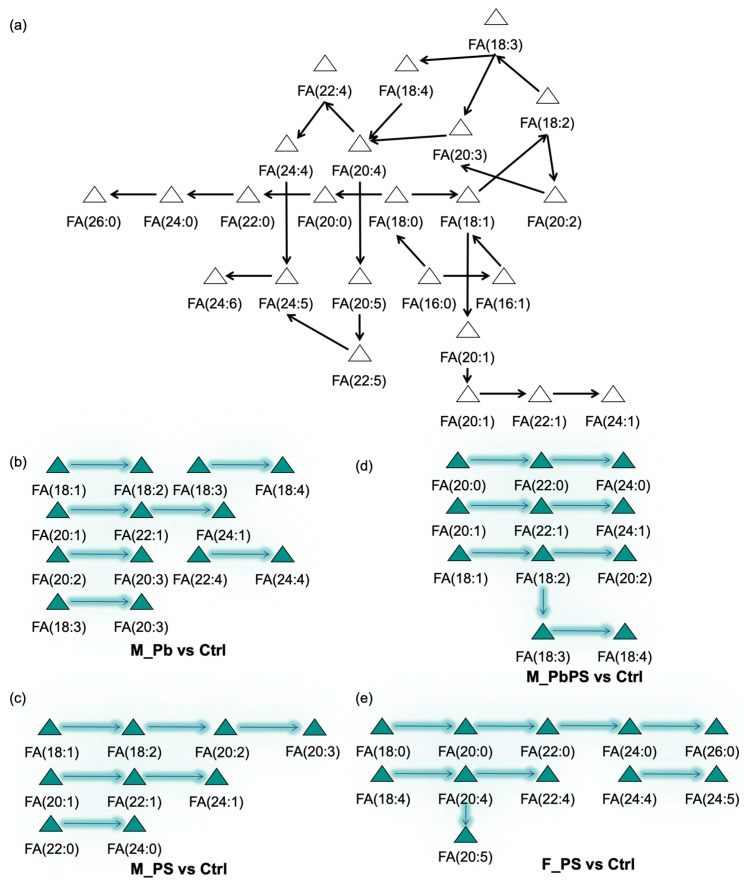
Activated fatty acid pathways by PbPS co-exposure. (**a**) Overview of fatty acid pathways. (**b**) Active pathways by Pb exposure in male mice. (**c**) Active pathways by PS exposure in male mice. (**d**) Active pathways by PbPS co-exposure in male mice. (**e**) Active pathways by PS exposure in female mice.

The overview of fatty acid pathways is shown in Figure 4a. In male mice, Pb individual exposure activated six fatty acid pathways, including FA(18:1) → FA(18:2), FA(18:3) → FA(18:4), FA(20:1) → FA(22:1) → FA(24:1), FA(20:2) → FA(20:3), FA(22:4) → FA(24:4), FA(18:3) → FA(20:3) (Figure 4b). Fatty acid pathways activated by PS individual exposure included FA(18:1) → FA(18:2) → FA(20:2) → FA(20:3), FA(20:1) → FA(22:1) → FA(24:1), FA(22:0) → FA(24:0) (Figure 4c). Fatty acid pathways activated by PbPS exposure included FA(20:1) → FA(22:1) → FA(24:1), FA(20:0) → FA(22:0) → FA(24:0), FA(18:1) → FA(18:2) → FA(20:2), FA(18:1) → FA(18:2) → FA(18:3) → FA(18:4) (Figure 4d). In female mice, PS individual exposure activated four fatty acid pathways, including FA(18:0) → FA(20:0) → FA(22:0) → FA(24:0) → FA(26:0), FA(18:4) → FA(20:4) → FA(22:4), FA(24:4) → FA(24:5), FA(18:4) → FA(20:4) → FA(20:5) (Figure 4e). Pb individual exposure or PbPS co-exposure did not activate any fatty acid pathways. Stronger responses in fatty acid metabolism were found in male mice compared with females.

### 3.5. Fatty Acid Pathways Suppressed by PbPS Co-Exposure

**Figure 5:**
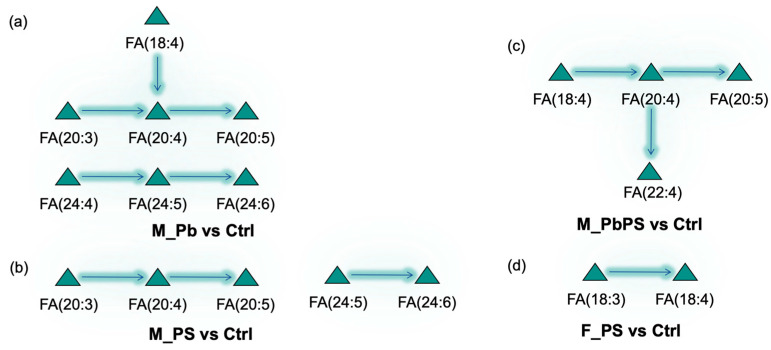
Suppressed fatty acid pathways by PbPS co-exposure. (**a**) Suppressed pathways by Pb exposure in male mice. (**b**) Suppressed pathways by PS exposure in male mice. (**c**) Suppressed pathways by PbPS co-exposure in male mice. (**d**) Suppressed pathways by PS exposure in female mice.

In male mice, Pb individual exposure suppressed several fatty acid pathways, including FA(18:4) → FA(20:4) → FA(20:5), FA(20:3) → FA(20:4) → FA(20:5), FA(24:4) → FA(24:5) → FA(24:6) (Figure 5a). Fatty acid pathways suppressed by PS individual exposure included FA(20:3) → FA(20:4) → FA(20:5), FA(24:5) → FA(24:6) (Figure 5b). Fatty acid pathways suppressed by PbPS co-exposure included FA(18:4) → FA(20:4) → FA(22:4), FA(18:4) → FA(20:4) → FA(20:5) (Figure 5c). In female mice, individual Pb or co-exposure of Pb and PS did not suppress any fatty acid pathways. FA(18:3) → FA(18:4) was suppressed by PS individual exposure in female mice (Figure 5d).

### 3.6. Lipid Pathways Activated by PbPS Co-Exposure

**Figure 6:**
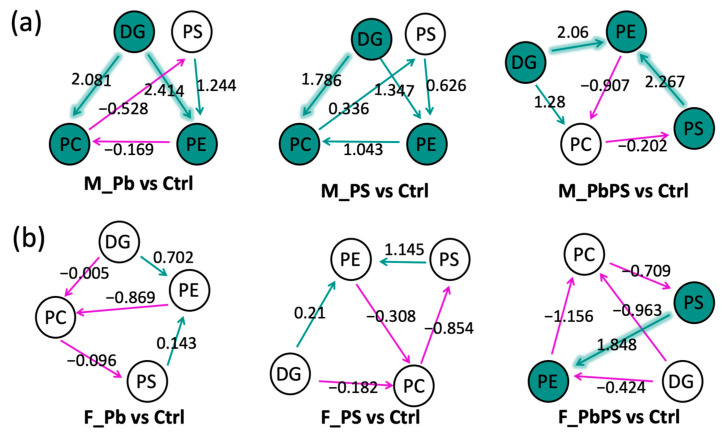
Activated lipid pathways by PbPS co-exposure. (**a**) Active pathways in male mice. Solid icon: Active status. Purple line: Negative Z-score. Green line: Positive Z-score. Highlighted green line: Positive Z-score implied in an active status. (**b**) Active pathways in female mice. Solid icon: Active status. Purple line: Negative Z-score. Green line: Positive Z-score. Highlighted green line: Positive Z-score implied in an active status. DG: diacylglycerol; PC: phosphatidylcholine; PE: phosphatidylethanolamine; PS: phosphatidylserine (note: PS in this figure represent lipids, rather than polystyrene MPs).

In addition to fatty acids, we further investigated the impact of PbPS co-exposure on complex lipid metabolism. In male mice, Pb individual exposure activated the transition from diacylglycerol (DG) to phosphatidylcholine (PC) and from DG to phosphatidylethanolamine (PE) (Figure 6a). The PS individual exposure group only activated the transition from DG to PC. PbPS co-exposure activated the transition from DG to PE and from phosphatidylserine to PE. In female mice, PbPS co-exposure activated the transition from phosphatidylserine to PE; meanwhile no lipid pathways were activated in individual exposure groups (Figure 6b). Consistent with previous findings, stronger responses were found in male mice compared with females. No lipid pathways were suppressed by either individual or co-exposure of Pb and PS MPs in both male and female mice.

## 4. Discussion

A large number of chemicals are present in the environment. Human and other living organisms are routinely exposed to a wide range of substances originating from diverse sources via multiple exposure routes. In the United States and European Union, assessment of the risks from chemical mixtures have been incorporated into regulatory frameworks [32]. Combined exposure to MPs and Pb is common, as both are widespread environmental pollutants and food-related chemicals. In addition, MPs could also function as vectors for heavy metals. Thus, a risk assessment of the combined exposure of MPs and Pb is a critical need. In this study, we investigated the effects of co-exposure to Pb and PS MPs on hepatic lipid metabolism in male and female C57BL/6 mice by integrating transcriptomics and untargeted lipidomics. We found that hepatic lipid metabolism was disrupted by the co-exposure of Pb and PS MPs [33]. Lipids play critical roles in energy metabolism, membrane stability and signaling. Disruption of lipid metabolism are linked to various diseases such as obesity, type 2 diabetes and metabolic syndrome [33].

Specific alterations in the lipidome were induced by the co-exposure of Pb and PS MPs, which were not observed in individual exposures. For instance, transition from phosphatidylserine to PE was only activated in the PbPS co-exposure group, rather than the individual exposure groups (Figure 6). There are four different pathways for PE synthesis in mammalian cells and each pathway of PE synthesis has independent and necessary functions, among which the CDP–ethanolamine pathway and the phosphatidylserine decarboxylation pathway are the most important pathways quantitatively [34]. Phosphatidylserine is biosynthesized in the membranes of endoplasmic reticulum and then transported to mitochondria where decarboxylation occurs to produce PE [34]. In addition, the transition from FA (18:2) to FA (18:3) was only activated by PbPS co-exposure rather than individual exposure in male mice (Figure 4), suggesting specific alterations were found for both complex lipids and fatty acids.

In HepG2 cells, Pb alone induced lipid accumulation by the regulation of autophagy process [35]. Pb exposure disturbed the lipid metabolism through the regulation of lipophagy process in microglia [36]. PS MPs alone disrupted hepatic lipid metabolism via the polarization of inflammatory Kupffer cell and IL-17/NF-κB signaling pathways [37]. Co-exposure to MPs and Pb could increase the accumulation of Pb in mice’s organs, which resulted in more severe toxicity [38]. In the Chinese mitten crab, PS MPs lead to the elevated bioaccumulation of Pb and aggravated Pb-induced oxidative damage and hepatopancreas injury [39]. Similarly, PS nanoplastics increased the deposition of Pb in the liver and exacerbated Pb-induced liver toxicity through the activation of oxidative stress in mice [40]. Increased accumulation of Pb in mice during the combinational exposure of Pb and MPs might also be a possible mechanism for the specific effects induced by the co-exposure in this study.

In the general population, males and females show different patterns in the distribution and lifelong trajectories of lipids and lipoproteins from infancy through adulthood [41]. Sex differences in circulatory lipids have been reported in previous studies [42,43]. Substantial differences in lipid metabolism were found between women and men, including the metabolism of dietary fatty acids, the secretion and clearance of very low-density lipoprotein-triglycerides, the rate of free fatty acid release from adipose tissue in relation to energy demands, and the extraction of free fatty acids from the bloodstream [44]. In the present study, the effects of co-exposure on liver transcriptome and lipidome were sex-specific. For instance, DG species were decreased by PbPS co-exposure in male mice but increased in female mice (Figure 3c,d). DG is unique in lipid metabolism because it functions both as a triglyceride synthesis intermediate and as a signaling molecule that activates DG-responsive proteins, thereby bridging energy metabolism and intracellular signaling [45]. DG has been reported to ameliorate non-alcoholic fatty liver and systemic lipid accumulation [46].

Untargeted lipidomic analysis showed that more lipids were significantly altered by PbPS co-exposure in male mice than in female mice (Figure 3c,d), which is consistent with the transcriptomics analysis. In addition, GSEA of liver transcriptome showed that fatty acid metabolism was downregulated by PbPS co-exposure only in male mice, rather than in female mice (Figure 2b,c). Furthermore, fatty acid pathway analysis also showed that some fatty acid pathways were active or suppressed in the PbPS co-exposure group of male mice, whereas no significant changes were found in female mice (Figure 4 and Figure 5). Pathway analysis of complex lipids also demonstrated that a stronger response to the co-exposure was found in male mice than in female mice, as there were two active pathways in males (DG to PE, phosphatidylserine to PE), whereas only one active pathway (phosphatidylserine to PE) was found in females by the co-exposure (Figure 6). Due to these conversions, increased level of PE species was found in PbPS co-exposure group in both male and female mice. Liver damage is associated with a low molar ratio of hepatic PC to PE [47,48,49]. Taken together, integrative analysis of transcriptomics and lipidomics showed that hepatic lipid metabolism was more sensitive to PbPS co-exposure in male mice than in female mice.

These sex differences observed in the PbPS co-exposure group might be attributable to the influences of sex hormones or sex-specific gene regulation. For instance, IL6/JAK/STAT3 signaling was upregulated in the co-exposure group in the male mice, whereas MYC targets V2 was upregulated in the female mice in the co-exposure group (Figure 2). The regulation of lipid homeostasis in the liver was empowered by mitochondria through the process of fatty acid oxidation and lipogenesis [50]. Notably, the mitochondria of females have greater resistance to oxidative damage than males [51], which might also explain why lipid metabolisms in male mice were more sensitive to PbPS co-exposure in the present study. These sex-specific responses need to be taken into consideration when investigating the toxicity of combined exposure to MPs and heavy metals.

This study has several limitations. First, the exposure period was only five weeks. Further studies with extended durations are needed to assess the long-term health impacts of the co-exposure. Second, quantification of PS MPs or Pb in the liver samples was not performed in present study. Future studies should include quantitative analysis to better understand the internal exposure.

## 5. Conclusions

This study investigated the impacts of PS MPs and lead co-exposure on hepatic lipid metabolism using transcriptomics and untargeted lipidomics. Hepatic lipid metabolism was disturbed by the co-exposure in C57BL/6 mice. These findings provide insights into the combined exposure risks of MPs and heavy metals. Future research is needed to investigate the long-term consequences, potential transgenerational effects, and intervention strategies to mitigate the combined toxicity of MPs and heavy metals.

## Data Availability

Data is available upon reasonable request.

## Abbreviations

The following abbreviations are used in this manuscript:

MPs	Microplastics
Pb	Lead
PS (microplastics)	Polystyrene
ALT	Alanine aminotransferase
MTBE	Methyl tert-butyl ether
MS/MS	Mass spectrometry
PLS-DA	Partial least squares discriminant analysis
GSEA	Gene set enrichment analysis
PE	Phosphatidylethanolamine
DG	Diacylglycerol
SM	Sphingomyelin
TG	Triacylglycerol
FAHFA	Fatty acyl esters of hydroxy fatty acid
PI	Phosphatidylinositol
PC	Phosphatidylcholine
FA	Fatty acid
Cer	Ceramide
BMP	Bis(monoacylglycero)phosphate
PG	1,2-diacyl-sn-glycero-3-phospho-(1’-sn-glycerol)
PS (lipids)	Phosphatidylserine
FC	Fold change
